# *In vitro* evaluation of graded level of Silkworm pupae (*Bombyx mori*) oil on methane production, fermentation characteristics, and protozoal populations

**DOI:** 10.14202/vetworld.2020.586-592

**Published:** 2020-03-28

**Authors:** G. Thirumalaisamy, Pradeep Kumar Malik, Atul P. Kolte, Raghavendra Bhatta

**Affiliations:** 1 ICAR-National Dairy Research Institute, Karnal, Haryana, India; 2 ICAR-National Institute of Animal Nutrition and Physiology, Adugodi, Bengaluru, Karnataka, India

**Keywords:** dry matter digestibility, methane production, protozoa, silkworm pupae oil

## Abstract

**Aim:**

The present study was undertaken to evaluate the effect of variable levels of silkworm pupae oil and roughage: concentrate ratio on *in vitro* methane production, fermentation characteristics, and rumen protozoa population.

**Materials and Methods:**

*In vitro* gas production study (24 h) was performed with graded levels of silkworm pupae oil, namely, 0.5, 1, 2, 4, and 5% of the basal diet and four variable dietary regimes consisting roughage and concentrate in different proportions (70:30, 60:40, 50:50, and 40:60). At the end of incubation, gas samples were analyzed for methane, while fermented rumen liquor was used for protozoa enumeration. A separate set of incubations was carried out for the determination of *in vitro* dry matter digestibility.

**Results:**

Results from the *in vitro* studies revealed no adverse impact of the silkworm pupae oil supplementation up to 2% level on total gas production. However, supplementation beyond 2% has shown a reduction in total gas production. Incubation with variable levels (0.5-5%) of silkworm pupae oil with different dietary regimes indicated negligible (3-5%) to a substantial reduction (25-30%) on methane production. A graded decrement in methane production was recorded with increasing levels of silkworm pupae oil. Similarly, the protozoal populations were decreased from 10 to 51.5% with graded levels of silkworm pupae oil in different dietary regimes as studies did not reveal any significant (p>0.05) variation between 2 and 4% of oil supplementation.

**Conclusion:**

The silkworm pupae oil supplementation at 2% level decreases methane production by 12-15% without any adverse impact on feed fermentation. Oil supplementation may have a more pronounced effect on methane reduction if added to high roughage diet at *in vitro* conditions. However, *in vivo*, studies in ruminants are warranted to confirm the methane reduction with silkworm pupae oil supplementation.

## Introduction

Climate change is one of the burning issues of recent time, and livestock production has been identified as one of the major driving factors. The impact of climate change is stratified depending on the agro-climatic ecoregions, demographic location, and resource richness. Since the 18^th^ century, various attempts have been made to understand the contributing factors and developing suitable ameliorative measures for minimizing the impact. Greenhouse gases (GHG) with high global warming potential lead to an acceleration in atmospheric temperature. GHG emit from the natural (wetlands, ocean, lakes and river water, termites, and other arthropods) and anthropogenic (fossil fuels, agriculture, waste water, landfilling, and biomass burning) sources which contribute 42 and 58%, respectively [1]. Among anthropogenic, agriculture is accountable for 13% (5.7 billion tons CO_2_ equivalent) of the emission and stands third after the energy and transport sector. Paddy cultivation and livestock production are two major components of the emission from agriculture, wherein livestock contributes 39% to the total GHG emissions [2,3].

Methanogenesis is an integral but wasteful process associated with enteric fermentation in the rumen. In addition to the driving factor in climate change, enteric methane emission also held responsible for the loss of biological energy. On an average, about 8-12% of the ingested feed energy is being wasted in the form of methane [4]. Thus, to correct production inefficiencies, it is desirable to reduce the methane emission from the livestock. Several options, including the use of plant secondary metabolites [5-9], organic acids, essential oils, and lipids, have been tried for the amelioration of methane emission. Oil feeding at an appropriate level is one of the promising strategies for enteric methane amelioration [10,11]., The high cost of conventional oil sources such as linseed, rapeseed, palm, and canola, restricts their use in the animal diet [12].

Pupa accounted for 60% of the cocoon weight and discarded as waste. Silkworm pupae oil has interesting properties of lowering cholesterol, improves memory [13], and anti-oxidation [14].

Silkworm pupae oil, due to its adequate availability and non-competition with human beings, could be an alternate source for reducing methane emission. In the present study, an attempt has been made to investigate the impact of graded levels of silkworm pupae on *in vitro* methane production when supplemented with the diet consist of variable proportions of roughage and concentrate.

## Materials and Methods

### Ethical approval

The study was not conducted on live animals; hence, no ethical approval was required.

### Location of the study

*In vitro* studies were conducted at the Energy Metabolism Laboratory of Bioenergetics and Environmental Sciences, Division of National Institute of Animal Nutrition and Physiology, Bengaluru, India.

### Experiment details

Feed ingredients were collected from the feed unit of the institute and grounded before formulating the basal diets. Concentrate mixture was prepared by the mixing of maize grain (40), soybean meal (35), wheat bran (22), mineral mixture (2), and salt (1). Basal diets were formulated with finger millet (*Eleusine coracana*) straw and concentrate in variable proportions. Details of various basal diets are given in Table-1. Five levels of silkworm pupae oil, namely, 0, 0.5, 1, 2, 4, and 5% were tested for the impact on methane production and feed fermentation with all the four basal diets (Table-2).

**Table-1:** Composition of basal diet combinations (on dry matter basis).

Basal diet	Finger millet straw	Concentrate mixture	Total
Diet 1 (HR)	70	30	100
Diet 2 (MC)	60	40	100
Diet 3 (ERC)	50	50	100
Diet 4 (HC)	40	60	100

HR=High roughage, MC=Medium concentrate, ERC=Equal roughage and concentrate, HC=High concentrate

**Table-2:** Different dietary regimes and silkworm pupae oil levels *in vitro* studies.

Diet	Oil level	Total diets
Diet 1 (HR)	0, 0.5, 1, 2, 4, and 5%	6
Diet 2 (MC)	0, 0.5, 1, 2, 4, and 5%	6
Diet 3 (ERC)	0, 0.5, 1, 2, 4, and 5%	6
Diet 4 (HC)	0, 0.5, 1, 2, 4, and 5%	6
		Total 24

HR=High roughage (R:C-70:30), MC=Medium concentrate (R:C-60:40), ERC=Equal roughage and concentrate (R:C-50:50), HC=High concentrate (R:C-40:60)

### Chemical composition

Finger millet straw, concentrate, and basal diets consisting variable proportions of straw and concentrate were analyzed for the proximate principles and fiber fractions as per AOAC, 2012 [15] (Method No. 934.01[moisture], 942.05 [total ash], 955.01 [total nitrogen], 920.39 [ether extract], 2002.04 [neutral detergent fiber], and 973.18 [acid detergent fiber]).

### *In vitro* incubation and gas analysis

The *in vitro* gas production technique of Menke and Steingass [16] was employed for the studies. Rumen fluid was collected from the cannulated Holstein bulls before morning (08.00 AM) feeding. The rumen content was collected in two fractions: The first solid portion (1/3^rd^) was collected in a polypropylene zip lock cover and the liquid fraction was collected in a screw cap conical flask (1 L). The solid, fluid fractions of the rumen contents were placed into a prewarmed thermos flask maintained at 39°C and transported to the laboratory for further processing. Thereafter, the rumen liquor was strained through four layers of muslin cloth and strained rumen liquor (SRL) was flushed with CO_2_ (UHP 99.99%, Chemix Pvt. Ltd.) until added to the rumen buffer medium solution.

About 200±10 mg of air-dried sample was weighed in a plastic boat with a removable stem and placed in the bottom of the syringe without sticking to the sides of the syringe. Silkworm pupae oil with varying levels of 0, 0.5, 1, 2, 4, and 5% was added to the basal diet as per the experimental plan. The syringes without feed and with standard hay were considered as blank and reference samples, respectively. Thereafter, Vaseline was applied in a thin layer without reaching the bottom of the piston and gently inserted. Each sample was prepared for the incubated with three replicates. The buffer medium was continuously bubbled with CO_2_. The blue color of the solution first changed to pink after adding freshly prepared reducing solution and finally became colorless. The required SRL was added into the media and allowed to mix properly for 10 min. Thereafter, 30 ml of incubation medium was dispensed into each syringe using semi-auto dispenser (Eppendorf, Varispenser® plus, 50 ml, Germany). The syringes were shaken gently, and the residual air or air bubble, if any, was removed, and subsequently, the outlet was closed. The initial position of the piston was recorded, and the syringes were placed in a water bath shaker at 39±0.5°C. After completion of 24 h of incubation, ice flakes were added to *in vitro* water bath to arrest the fermentation. The final reading was recorded by seeing the piston position. Total gas produced (ml/200 mg DM) during 24 h of incubation was measured through the piston displacement method. The total gas production was calculated by subtracting the initial piston position from the final. A correction for the gas production in corresponding blanks was also considered. The total gas production was expressed in ml/200 mg DM as well as in ml/g DM.

“Total gas production (ml/200 mg) = gas production in sample-blank”


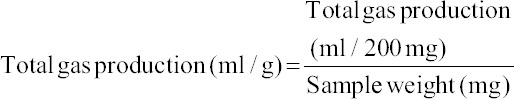


After 24 h of incubation, gas samples were collected in a pre-vacuumed vial and 1.0 ml of gas sample was withdrawn in an air-tight syringe (Hamilton Company, 1 ml Reno, NV, USA) and injected manually into a gas chromatograph (Chemito, GC1000, Thermo Fisher Scientific) equipped with thermal conductivity detector and packed column Porapak-Q.

The injector, oven, column, and detector temperatures for conditioning were maintained as 120, 150, and 230°C, respectively, for 60 min; thereafter, the temperature was changed to 60, 100, and 160°C, respectively, for analyzing methane gas. The flow rate of carrier gas (N_2_) through the column was maintained as 40 ml/min. The standard of methane (Chemix Specialty Gases, Bengaluru) was injected twice before and after analyzing the actual gas samples. The peak of methane was identified based on the retention time of standard gas, and the area was employed to calculate the methane percentage in the gas sample.


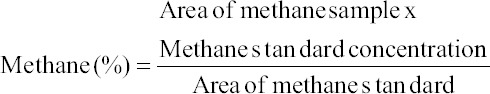


Based on the % methane in gas sample, methane volume was calculated as follows


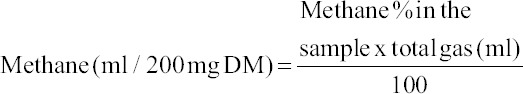



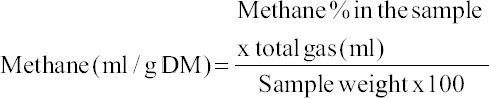


### Protozoal enumeration

The fermented rumen liquor samples were gently mixed by inversion and 1 ml of sample added with 2 ml formalin solution (37% w/v). The samples were enumerated for their ciliate protozoal count on the same day. The rumen ciliates were identified according to the Hungate [17]. The hemocytometer (Neubauer, Germany) chamber had a depth of 0.1 mm. The protozoa numbers were calculated, as described by Kamra *et al*. [18],


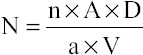


Where,

N-Number of protozoa per ml of rumen liquor

n-Average number of protozoa per microscopic field

A-Area of slide on which the diluted sample of rumen liquor is spread

D-Dilution of rumen liquor

a-Area of the microscopic field

v-Volume of diluted rumen liquor in the cavity.

### *In vitro* dry matter digestibility (IVDMD)

The method of Goering and Van Soest [19] was used for determining the IVDMD experiments and performed a separate set of incubations through *in vitro* gas production test (24 h) for the determination of IVDMD. About 500 mg was taken in Hohenheim syringes (Haberle, 100 ml, Germany) and made ready for the incubation. A portion (40 ml) of the rumen-fluid medium was transferred to each syringe and incubated in a water bath at 39°C for 24 h duration. After incubation, entire content in the syringe was poured to the fiber bags, and the syringe was rewashed until the complete removal of residual contents and poured in fiber bags. The neutral detergent fiber estimation was performed as per AOAC [15], (Method No. 2002.04). The following equation was used to determine IVDMD.


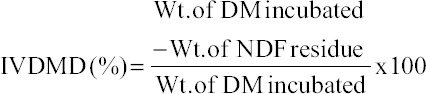


### Statistical analysis

Data from the *in vitro* studies were analyzed using one-way ANOVA in SPSS (version 21) [20]. Mean separations with a significant F (p<0.05) for the effect of oil supplementation were compared. Differences between means were compared by Tukey’s method and considered significant at p<0.05. The results are presented as means with a standard error of mean and p-value. Superscriwpts have been placed wherever means are significant at 5% and 1% [21].

*Y_ij_* = *μ*+*α_i_*+*ε_ij_*

Yij=Effect, µ=Mean of i-th level, α_i_=Oil levels (i=0, 0.5, 1, 2, 4, and 5), εij=Error term.

## Results

### Chemical composition and total gas production

Chemical composition of finger millet straw, concentrates, and basal diets are presented in Table-3. Total gas production as influenced by the graded levels of silkworm pupae oil supplementation (0.5, 1, 2, 4, and 5%) and dietary regimes (variable proportion of finger millet straw and concentrate) is given in Table-4. Results from the *in vitro* studies indicated a variable response of graded levels of silkworm pupae oil supplementation on total gas production (ml/g). Silkworm pupae oil supplementation with roughage-to-concentrate ratio at 70:30 finger millet straw and concentrate revealed a significant (p<0.05) reduction in total gas production with oil supplementation beyond 1% of the basal diet. However, a slight increase in concentrate proportion (up to 40%) did not reveal any reduction in total gas production with oil supplementation up to 2%. In contrary, an increment in oil supplementation at 4% decreased (p<0.05) total gas production with the basal diet consisted of 40% concentrate. These results established that the oil supplementation at a higher level (≥4%) has an adverse impact on feed fermentation evident with decreased total gas production. Various treatments with 50:50 of finger millet straw and concentrate based basal diets also revealed a similar trend. Further increase in the proportion of concentrate (60%) in the basal diet led to a decrease (p<0.05) in total gas production even at 2% silkworm pupae oil supplementation.

**Table-3:** Chemical composition (g/100g DM) of roughage, concentrate, and basal diets.

Item	OM	CP	EE	NDF	ADF	TA
Finger millet straw	87.6	3.76	1.35	76.2	54.7	12.4
Concentrate	92.7	20.5	3.03	50.4	12.9	7.30
Concentrate ingredients						
Maize	98.6	9.82	5.05	50.6	9.23	1.40
Soybean meal	91.3	43.9	1.29	51.1	26.5	8.70
Wheat bran	95.3	12.1	2.99	50.2	14.5	4.70
Basal diet						
Diet 1 (HR)	89.3	10.5	1.83	67.7	43.8	10.7
Diet 2 (MC)	89.7	12.3	1.96	64.4	39.4	10.3
Diet 3 (ERC)	90.4	13.8	2.13	60.8	35.1	9.60
Diet 4 (HC)	91.0	15.4	2.26	59.8	30.0	9.00

DM: Dry matter, OM=Organic matter, CP=Crude protein (N_2_ × 6.25), EE=Ether extract, NDF=Neutral detergent fiber, ADF=Acid detergent fiber, TA=Total ash, HR=High roughage, MC=Medium concentrate, ERC=Equal roughage and concentrate, HC=High concentrate

**Table-4:** Effect of silkworm pupae oil supplementation at graded levels with different dietary regimes on total gas production.

Treatment	Total gas (ml/g DM)			

	HR	MC	ERC	HC
T_0_	186^c^	196^b^	217^c^	225^d^
T_1_	182^bc^	193^b^	214^c^	223^d^
T_2_	179^ab^	189^ab^	213^bc^	217^cd^
T_3_	177^ab^	187^ab^	208^abc^	213^bc^
T_4_	175^a^	181^a^	204^ab^	208^ab^
T_5_	174^a^	180^a^	202^a^	205^a^
SEM	0.841	1.25	1.15	1.27
p-value	<0.001	<0.001	<0.001	<0.001

HR=High roughage, MC: Medium concentrate, ERC=Equal roughage and concentrate, HC=High concentrate. T_0_=Basal diet (control-no oil), T_1_=Silkworm pupae oil supplementation at 0.5% of basal diet, T_2_=Silkworm pupae oil supplementation at 1% of basal diet, T_3_=Silkworm pupae oil supplementation at 2% of basal diet, T_4_=Silkworm pupae oil supplementation at 4% of basal diet and T_5_=Silkworm pupae oil supplementation at 5% of basal diet. SEM=Standard error of mean, Mean values bearing different superscripts in a column differ significantly (p<0.05)

### *In vitro* methane production

Data pertaining to the impact of variable levels of silkworm pupae oil supplementation are presented in Table-5. A reduction of 5-29% in methane production with graded supplementation of silkworm pupae oil was recorded in the present study. Results have indicated a progressive decrease in methane production (ml/g DM) with increasing levels of oil supplementation in the basal diet. Silkworm pupae oil supplementation at 2% or higher led to a significant (p<0.05) reduction in methane production with all the basal diets formulated with variable proportions of roughage and concentrate.

**Table-5:** Effect of silkworm pupae oil supplementation at graded levels with different dietary regimes on methane production.

Treatment	Methane (ml/g DM)				Methane reduction (%)			
	
	HR	MC	ERC	HC	HR	MC	ERC	HC
T_0_	28.5^e^	33.7^e^	40.7^e^	43.4^e^	-	-	-	-
T_1_	27.0^de^	32.6^de^	38.8^de^	41.5^de^	5.28	3.30	4.63	4.52
T_2_	25.1^cd^	30.5^cd^	38.0^cd^	39.6^cd^	11.8	9.36	6.72	8.88
T_3_	23.4^bc^	28.6^bc^	36.5^bc^	38.4^bc^	18.0	15.0	10.4	11.7
T_4_	22.4^ab^	26.7^ab^	34.6^ab^	35.8^ab^	21.4	20.8	14.9	17.6
T_5_	20.2^a^	25.9^a^	33.7^a^	34.6^a^	29.2	23.1	17.2	20.4
SEM	0.427	0.428	0.374	0.465	-	-	-	-
p-value	<0.001	<0.001	<0.001	<0.001	-	-	-	-

T_0_=Basal diet (control-no oil), T_1_=Silkworm pupae oil supplementation at 0.5% of basal diet, T_2_=Silkworm pupae oil supplementation at 1% of basal diet, T_3_=Silkworm pupae oil supplementation at 2% of basal diet, T_4_=Silkworm pupae oil supplementation at 4% of basal diet, and T_5_=Silkworm pupae oil supplementation at 5% of basal diet. SEM=Standard error of mean, Mean values bearing different superscripts in a column differ significantly (p<0.05)

### IVDMD

Results with graded levels of silkworm pupae oil and dietary regimes revealed a differential response for DMD. Results indicated that there was an increase in DMD with the increasing levels of concentrate above 30% of the basal diet. DMD in treatment T_1_-T_5_ was decreased due to silkworm pupae oil supplementation at graded levels. The decrease in DMD at oil supplementation beyond 2% level was significant as compared to control treatment (Table-6).

**Table-6:** Effect of graded levels of silkworm pupae oil supplementation with different dietary regimes on *in vitro* dry matter digestibility (%).

Treatment	Dry matter digestibility (%)			

	HR	MC	ERC	HC
T_0_	62.1^d^	68.1^c^	72.4^c^	74.0^c^
T_1_	61.2^cd^	67.2^bc^	71.3^bc^	73.3^bc^
T_2_	60.3^bc^	66.7^b^	71.1^bc^	73.3^bc^
T_3_	59.6^b^	66.3^b^	70.1^ab^	73.0^bc^
T_4_	59.4^b^	64.8^a^	70.0^ab^	72.6^ab^
T_5_	57.4^a^	64.2^a^	68.8^a^	72.0^a^
SEM	0.268	0.255	0.247	0.139
p-value	<0.001	<0.001	<0.001	<0.001

HR=High roughage, MC=Medium concentrate, ERC=Equal roughage and concentrate, HC=High concentrate, T_0_=Basal diet (control-no oil), T_1_=Silkworm pupae oil supplementation at 0.5% of basal diet, T_2_=Silkworm pupae oil supplementation at 1% of basal diet, T_3_=Silkworm pupae oil supplementation at 2% of basal diet, T_4_=Silkworm pupae oil supplementation at 4% of basal diet, and T_5_=Silkworm pupae oil supplementation at 5% of basal diet. SEM=Standard error of mean, Mean values bearing different superscripts in a column differ significantly (p<0.05)

### Protozoal population

The present study indicated a variable response of oil supplementation on rumen protozoa (Table-7). A reduction of 8-51% in rumen protozoal population (×10^6^/ml) was recorded with variable proportions of roughage, concentrate, and silkworm pupae oil in the basal diets. Silkworm pupae oil supplementation at 2% of the basal diet revealed an average reduction of 20-34% in the rumen protozoal population.

**Table-7:** Effect of silkworm pupae oil supplementation at graded levels with different dietary regimes on total protozoal population (×10^6^/ml).

Treatment	Total protozoa				Reduction (%)			
	
	HR	MC	ERC	HC	HR	MC	ERC	HC
T_0_	4.86^b^	5.40^d^	6.16^d^	5.65^d^	-	-	-	-
T_1_	4.36^ab^	4.93^cd^	5.30^cd^	4.75^cd^	10.3	8.55	13.9	15.9
T_2_	4.14^ab^	4.51^bcd^	4.53^bc^	4.17^bc^	14.8	16.4	26.4	26.2
T_3_	3.82^ab^	4.32^abc^	4.06^ab^	3.97^bc^	21.4	20.0	34.1	29.6
T_4_	3.24^a^	3.84^ab^	3.73^ab^	3.50^ab^	33.4	28.8	39.4	38.0
T_5_	3.09^a^	3.42^a^	3.15^a^	2.74^a^	36.5	36.6	48.8	51.5
SEM	0.170	0.144	0.190	0.176	-	-	-	-
p-value	0.011	<0.001	<0.001	<0.001	-	-	-	-

HR-High roughage, MC=Medium concentrate, ERC=Equal roughage and concentrate, HC=High concentrate, T_0_=Basal diet (control-no oil), T_1_=Silkworm pupae oil supplementation at 0.5% of basal diet, T_2_=Silkworm pupae oil supplementation at 1% of basal diet, T_3_=Silkworm pupae oil supplementation at 2% of basal diet, T_4_=Silkworm pupae oil supplementation at 4% of basal diet, and T_5_=Silkworm pupae oil supplementation at 5% of basal diet. SEM=Standard error of mean, mean values bearing different superscripts in a column differ significantly (p<0.05)

## Discussion

Silkworm pupae oil consists of α-linolenic, oleic, linoleic, palmitic, and palmitoleic fatty acids. More than two-third of total fatty acids in silkworm pupae oil are unsaturated fatty acids [13]. Polyunsaturated fatty acids act through the inhibition of H_2_ producers such as protozoa in the rumen [22]. Results from the present *in vitro* study indicated that the silkworm pupae oil supplementation decreased total gas, methane production, and rumen protozoal population. Similar to the present results, a linear decrease in total gas production was reported with graded supplementation of coconut oil [23]. Pilajun and Wanapat [24] reported that supplementation of coconut and almond oil either alone (5%) or combination (50:50) at 5% with variable roughage and concentrate ratio revealed variable response on gas production. The coconut oil (8%) supplementation at a higher level decreased the total gas production [25]. In contrast, no reduction in total gas production was observed with supplementation of 3% fish oil [26]; 3% coconut oil [27]; and 2% sunflower oil alone or in combination with fish oil (1%) [28]. Oil supplementation beyond optimum level has an adverse impact on the feed fermentation, as evidenced in present study and thereby affects gas production. However, the adverse impact is dose-dependent and supplementation of 2% silkworm pupae oil *per se* did not affect the digestibility. The results are in agreement with the previous findings [24, 27-30], reported a significant reduction in methane production with oil supplementation. Unsaturated fatty acids in silkworm pupae oil could be toxic to the protozoa, as evidenced in the present study with their decreasing numbers. In the present study, we have not explored the impact of oil supplementation on methanogens; nevertheless, the unsaturated fatty acids are well known for their methanogens toxicity [31].

Keeping the results from this study in view, supplementation of silkworm pupae oil at 2% to achieve a 12-15% reduction in methane production without compromising the DMD appears promising. Results in this study indicated comparatively more methane reduction with oil silkworm pupae oil supplementation in high roughage diet than of more concentrated. Fermentation of fibrous feed produces more methane as compared to concentrate as the later one is rich in soluble carbohydrates. Thus, the oil supplementation modulates methane production when added to such diet, which has more intensity of methane-producing substances (fiber). The study of Kongmun *et al*. [25] also reported a decrease in DMD with increasing levels of coconut oil in the diet. This reduction in fiber digestibility was attributed to the interaction between microbes and fatty acids, whereas fatty acids may adsorb either onto microbes or feed particles that interact with dietary fiber [32]. Lipids are quite effective as defaunating agents [33], as they reduce the protozoal population and further decrease methane production. It is clearly confirmed from our study that the significant reduction of ≥20% in the rumen protozoa with a level of ≥2% of oil with various basal diets could be accountable for the reduction in methane production. However, at higher levels (beyond 2%), the reduction in DMD with a concurrent reduction in rumen protozoa appears to be equally responsible for the reduction of methane production. The results are in accordance with the previous studies, where reported the reduction in the protozoal population with oil supplementation [27, 34-36].

## Conclusion

From the *in vitro* studies, it can be inferred that the silkworm pupae oil supplementation at 2% level decreases methane production by 12-15% without any adverse impact on feed fermentation. Oil supplementation may have a more pronounced effect on methane reduction if added to a high roughage diet. However, *in vivo*, studies in ruminants are warranted to confirm the methane reduction with silkworm pupae oil supplementation.

## Authors’ Contributions

This study was a part of the Ph.D. thesis of the first author, GT, who carried out the research under the guidance of RB and PKM. The article was drafted by GT and PKM. The revision was made by RB, PKM, and APK. All authors have read and approved the final version of the manuscript.
